# Supplementary material for: Effects of Flight Restraint and Housing Conditions on Feather Corticosterone in White Storks Under Human Care
Source: Animals (Basel). 2025 Jun 25;15(13):1878. doi: 10.3390/ani15131878 (PMC12248471; doi:10.3390/ani15131878)
Supplement: Supplementary file 1 [file animals-15-01878-s001.zip › QuestionaireStork.pdf]

Fragebogen Weißstörche 2022

Fragenbogen im Rahmen der Studie

„Der Einfluss der Flugunfähigkeit auf das Tierwohl von Weißstörchen (*Ciconia ciconia*) durch Bestimmung von Corticosteron aus Federproben und der Analyse des Verhaltens“

Spezies: Weißstorch (*Ciconia ciconia*)

Zoo: Ansprechpartner/in:

Anzahl der flugunfähigen Tiere: Anzahl der flugfähigen Tiere:

1. Methoden des Flugunfähigmachens

	Armschwingen gestutzt	kupiert	Federfollikel extipiert	Verödung des germinativen Gewebes (Laser, Kryosonde, etc.)	verunfallt
Identifikation der Tiere (Ringnr., etc.)					

Sonstiges: ____

2. Gehege

	Fläche (in m^2)	Volumen (in m^3) (*Höhe des Dachs x Fläche)*
bitte in dieser Zeile eintragen:		

Fragebogen Weißstörche 2022

3. Management

	Vergesellschaftung	ja	nein	mit Tierart:
bitte in dieser Zeile ankreuzen:				
	nächtliche Aufstallung	ja	nein	mit Tierart:
bitte in dieser Zeile ankreuzen:				

4. Erkrankungen

	Infektionskrankheit	Akut (< 2 Wochen)	Chronisch (> 2 Wochen)
bitte in dieser Zeile Anzahl der Tiere angeben:			
	Unfall/ Verletzung	Akut (< 2 Wochen)	Chronisch (> 2 Wochen)
bitte in dieser Zeile Anzahl der Tiere angeben:			
	Fußgesundheit – Schweregrad (o.b.B., ggr., mgr., hgr.)	Beispiel: Sohlengeschwür, Risse	
Identifikation der Tiere	*Bsp.: A7W678: mgr.*	*Sohlengeschwür*	

5. Fütterung

	Frequenz	1-2 x tägl.	> 2 x tägl.
bitte in dieser Zeile eintragen:			
	Fischmenge	Fischart	Sonstiges
bitte in dieser Zeile eintragen:			
	Partnerfütterung	ja	nein
bitte in dieser Zeile ankreuzen:			

Bemerkungen:

Die nächsten Zeilen können Sie/ könnt Ihr gerne für individuelle Bemerkungen nutzen:

____

____

____

____

____

Bei Fragen oder Anmerkungen können Sie sich/ könnt ihr euch gerne bei mir melden:

frederike.liermann@gmail.com [redacted]

Vielen Dank nochmals für Ihre/ Eure Unterstützung und Teilnahme!

Frederike Liermann (Doktorandin)
