# Supplementary material for: Effects of Flight Restraint and Housing Conditions on Feather Corticosterone in White Storks Under Human Care
Source: Animals (Basel). 2025 Jun 25;15(13):1878. doi: 10.3390/ani15131878 (PMC12248471; doi:10.3390/ani15131878)
Supplement: Supplementary file 1 [file animals-15-01878-s001.zip › Supplementary Material.docx]

Supplementary Material: Violin Plots of Behavioural Observations


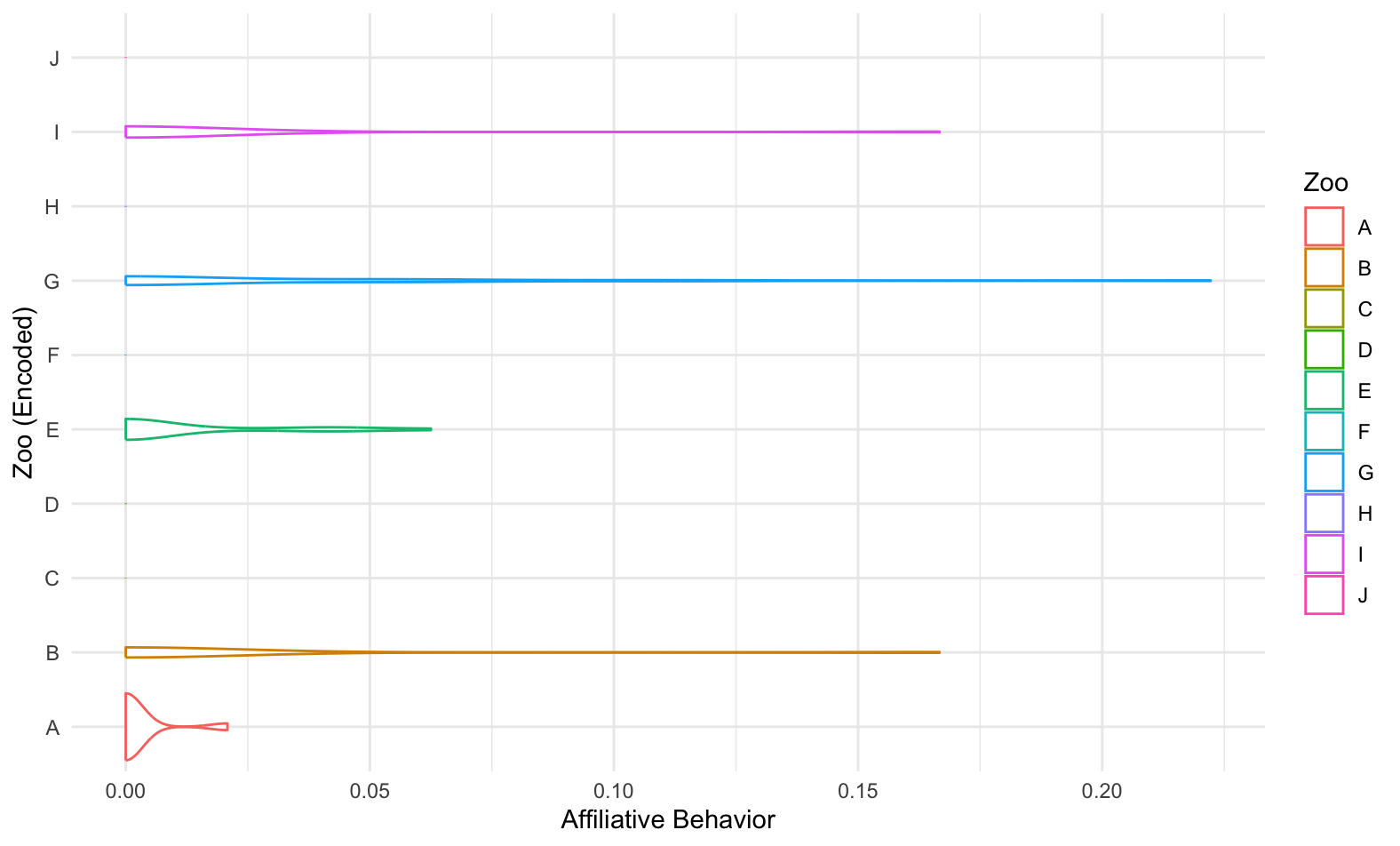


**Figure S1. Relative proportion of affiliative behaviour (% of total observed activity) across zoos.**
Violin plots show the percentage of total observed behaviour spent walking in white storks across ten zoos (A–J). The y-axis shows anonymized zoo identifiers, while the x-axis indicates walking behaviour as a proportion (%) of total activity recorded during behavioural observations.


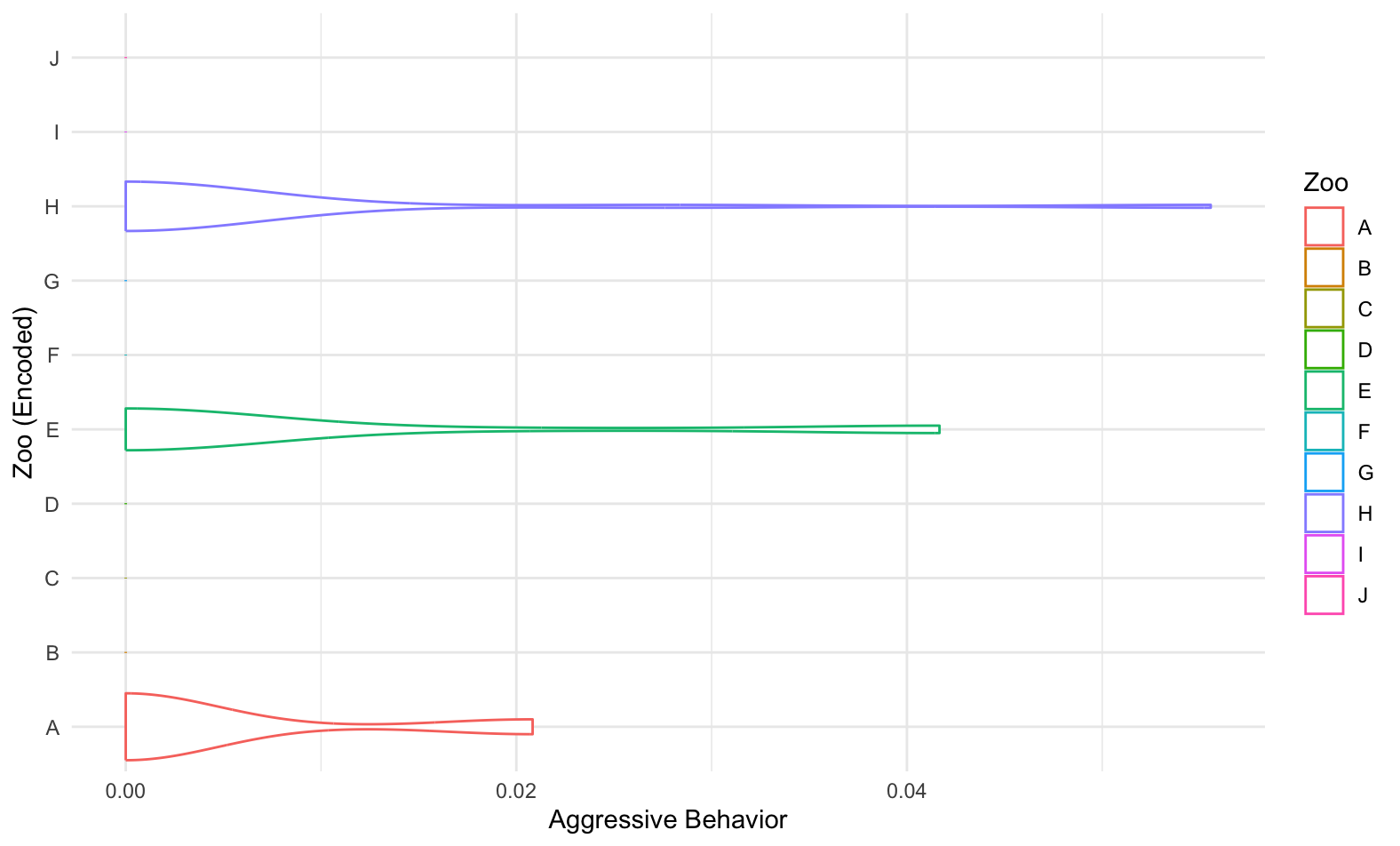


**Figure S2. Distribution of aggressive behaviour across zoos.**
Violin plots display the proportion of aggressive behaviours observed per zoo (A–J).


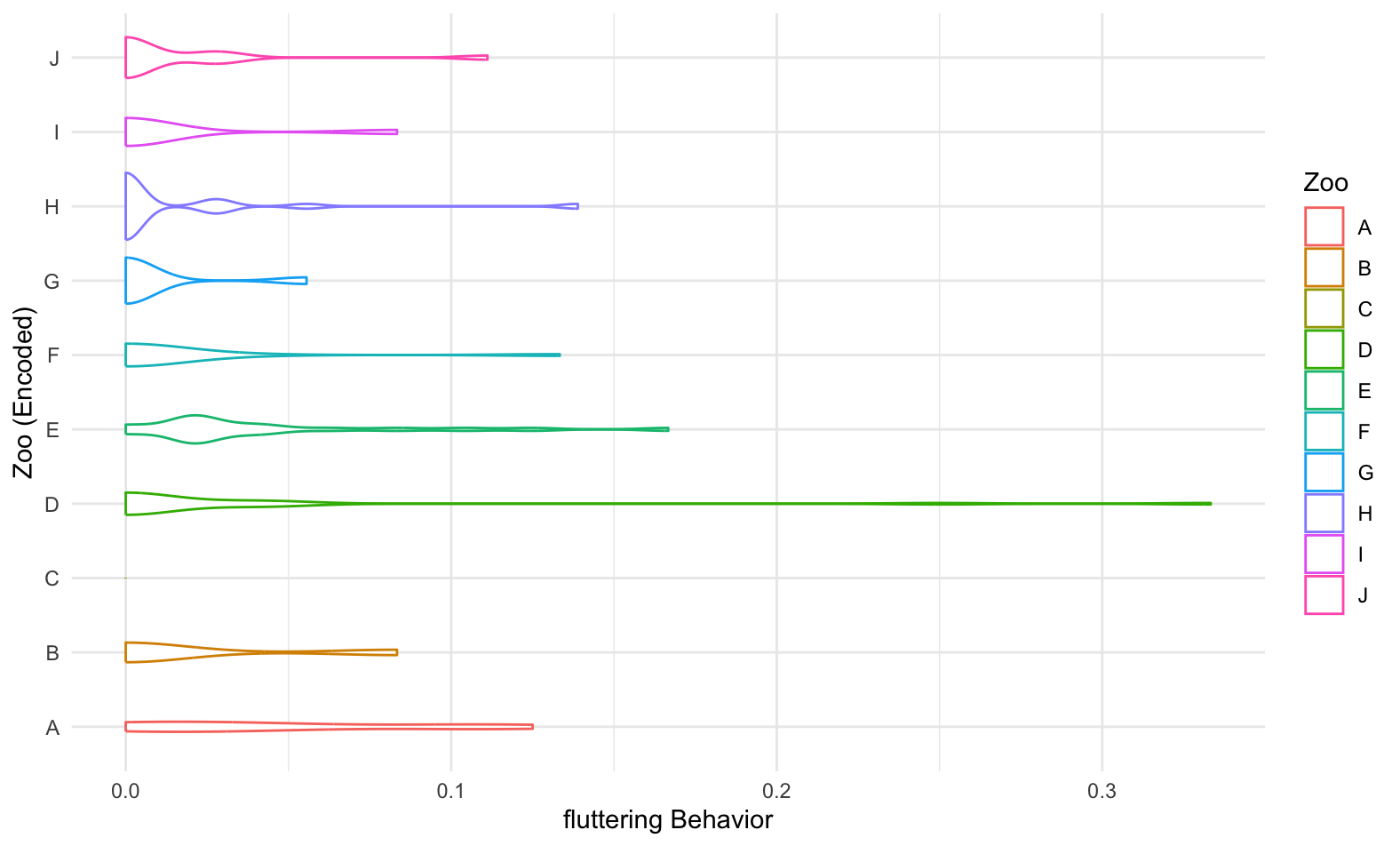


**Figure S3. Distribution of fluttering behaviour across zoos.**
Violin plots display the proportion of fluttering behaviours observed per zoo (A–J).


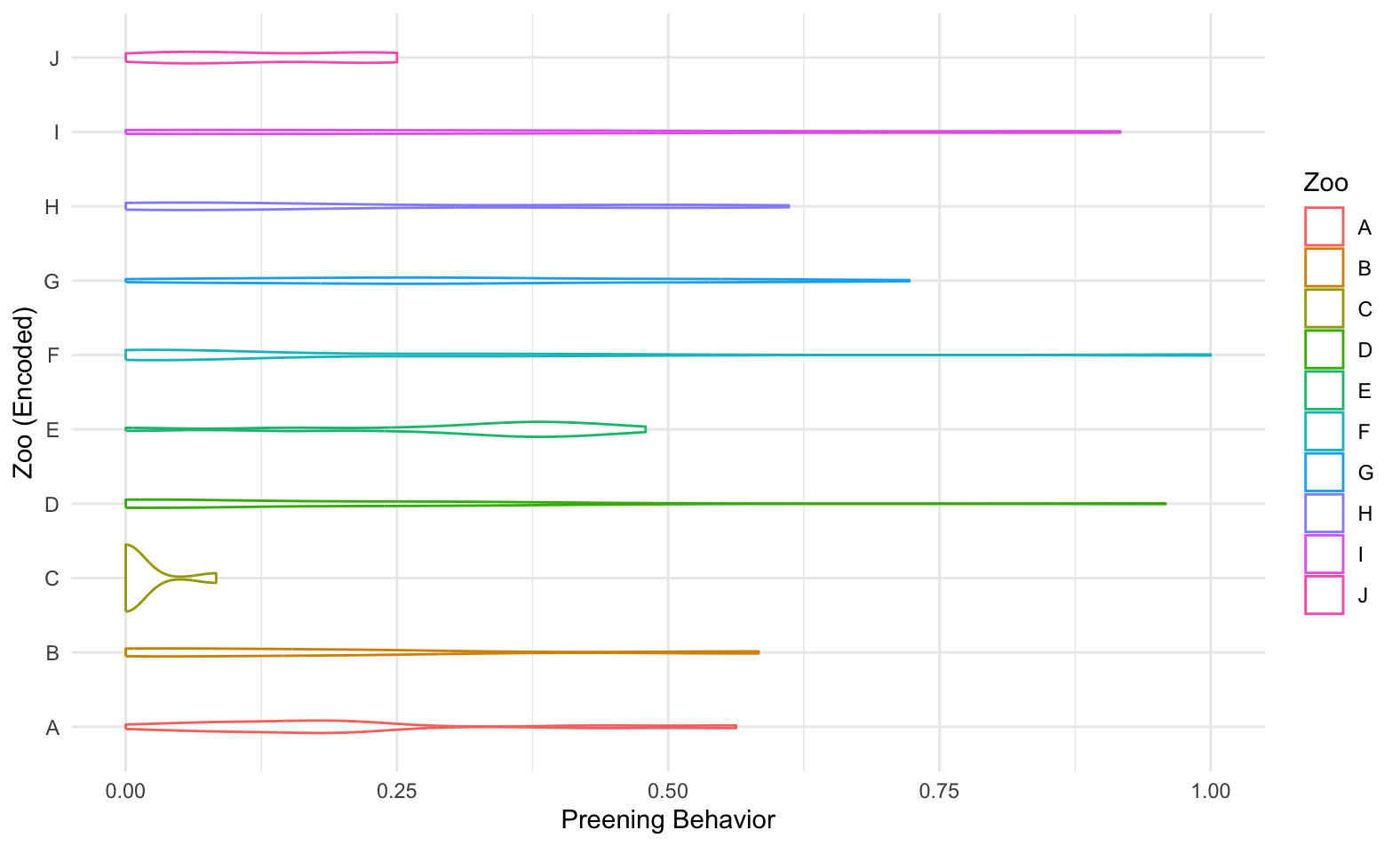


**Figure S4. Distribution of preening behaviour across zoos.**
Violin plots display the proportion of preening behaviours observed per zoo (A–J).


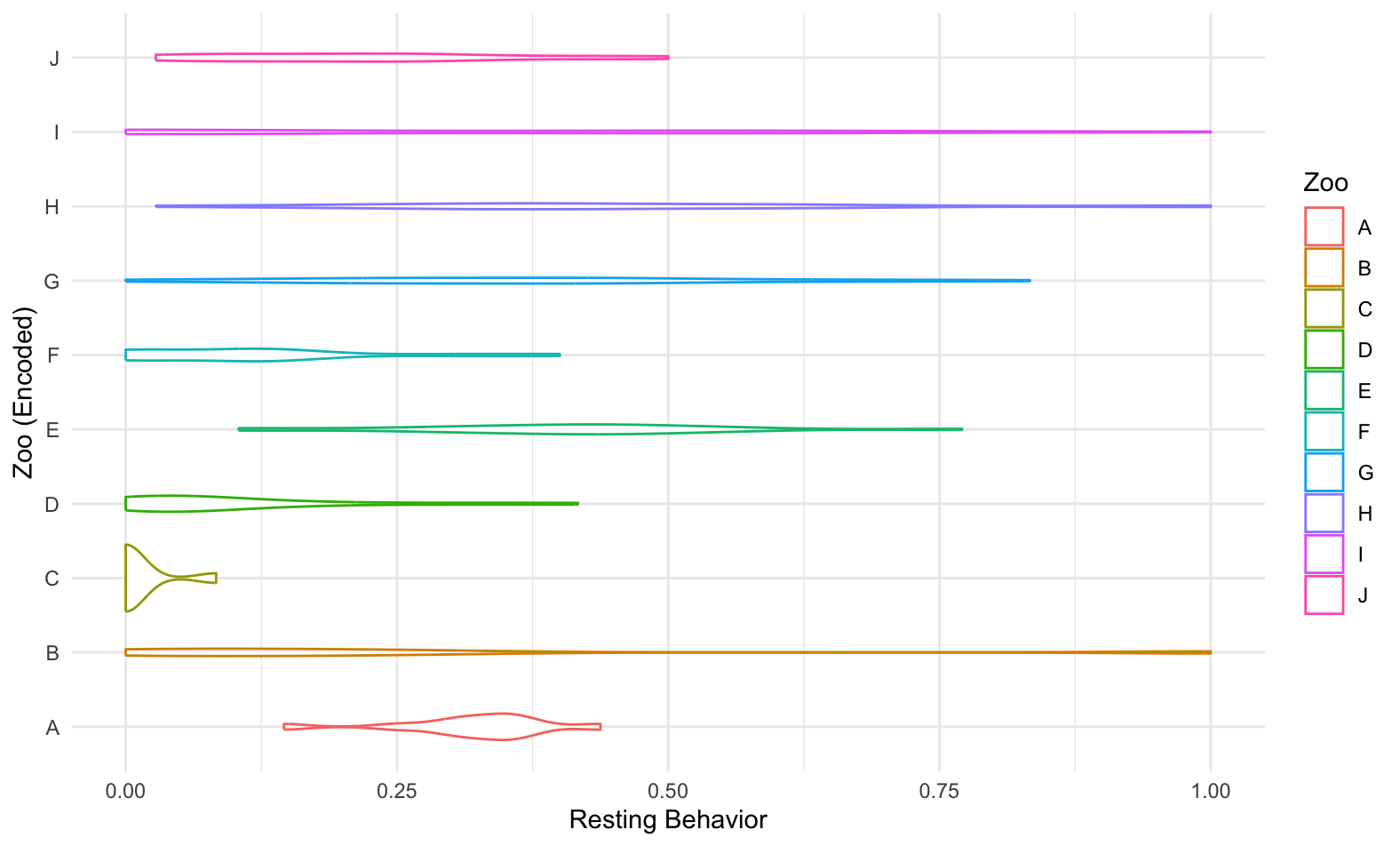


**Figure S5. Distribution of resting behaviour across zoos.**
Violin plots display the proportion of resting behaviours observed per zoo (A–J).


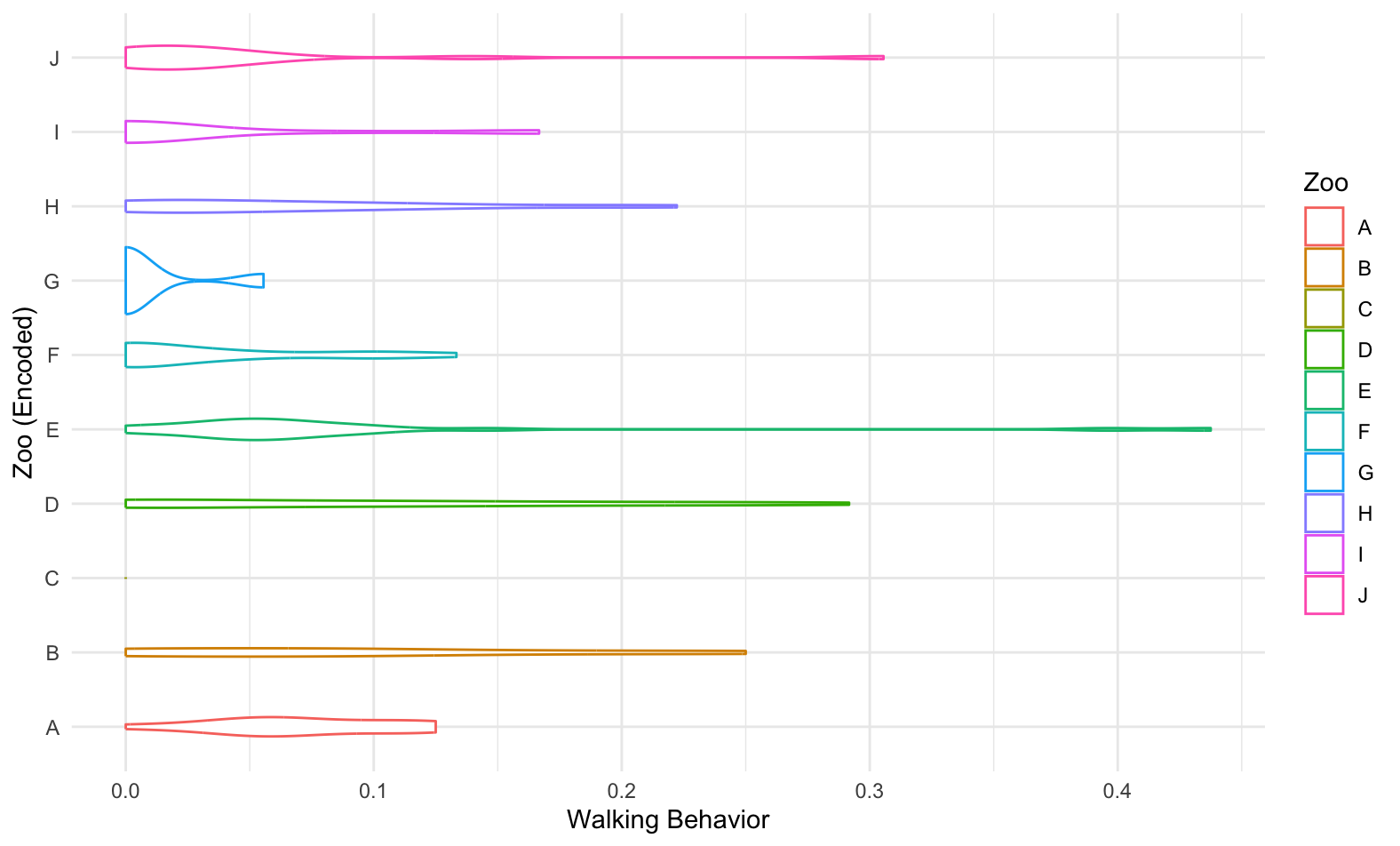


**Figure S6. Distribution of walking behaviour across zoos.**
Violin plots display the proportion of walking behaviours observed per zoo (A–J).
